# An Unusual Presentation of Secondary Hemophagocytic Lymphohistiocytosis With Melena: A Case Report

**DOI:** 10.7759/cureus.100641

**Published:** 2026-01-02

**Authors:** Saket Kant, Pradeep Tomar, Swapnil Gupta, Chhaya Yadav, Sparsh Rawat

**Affiliations:** 1 Internal Medicine, Graphic Era Institute of Medical Sciences, Dehradun, IND; 2 Nephrology, Topiwala National Medical College and Bai Yamunabai Laxman (BYL) Nair Charitable Hospital, Mumbai, IND

**Keywords:** bone marrow hemophagocytosis, case report, hemophagocytic lymphohistiocytosis (hlh), hyperferritinemia, secondary hlh

## Abstract

Hemophagocytic lymphohistiocytosis (HLH) is a rare, life-threatening hyperinflammatory syndrome characterized by excessive immune activation leading to multiorgan involvement. We present a case of an adult male with secondary HLH triggered by typhoid infection, manifesting with persistent fever, cytopenias, hepatosplenomegaly, and bone marrow hemophagocytosis. This report details the clinical presentation, laboratory findings, diagnostic challenges, management, and outcome, underscoring the importance of timely diagnosis and corticosteroid therapy. The clinical course and investigations fulfilled the established diagnostic criteria, illustrating the complexities of differentiating HLH from sepsis and other mimickers. Treatment with high-dose methylprednisolone led to clinical and hematological recovery. This case aligns with literature emphasizing early recognition and immunosuppressive therapy for improved prognosis in secondary HLH.

## Introduction

Hemophagocytic lymphohistiocytosis (HLH) is a life-threatening syndrome of severe immune dysregulation. It may be primary (genetic) or secondary to infections, malignancies, or autoimmune diseases [1,2]. Regardless of the trigger, HLH is characterized by uncontrolled activation of lymphocytes and macrophages, leading to a hyper-inflammatory state, cytokine storm, and progressive tissue damage [3]. The diagnosis is challenging due to a nonspecific presentation overlapping with infections and sepsis. Prompt recognition and immunosuppressive treatment are crucial to prevent mortality [4]. This case illustrates secondary HLH triggered by typhoid fever in an adult patient presenting with melena, emphasizing the need for prompt fulfillment of diagnostic criteria and treatment response.

## Case presentation

A 45-year-old male with a 20-year history of well-controlled type 2 diabetes mellitus presented initially with a seven-day history of fever and a two-day history of melena. Initially, the patient was admitted under the care of Gastroenterology in view of melena, where he underwent UGI endoscopy, which showed mucosal bleeding spots. Blood investigations revealed severe thrombocytopenia with a platelet count of 25000/µL. The patient received eight units of Random donor platelets (RDP) in view of ongoing melena with severe thrombocytopenia. However, upon continuous bleeding and no improvement in platelets, he was referred to our institute, a tertiary care center. On initial evaluation, the patient exhibited pallor and petechial rash on both lower limbs. Physical examination revealed hepatomegaly (4 cm below the right costal margin) and splenomegaly (3 cm below the left costal margin). CBC showed anemia with further fall in platelets (platelet count 2000/µL). Vital signs were stable with no neurological deficits. The patient was immediately shifted to the ICU, where he developed a fever with a temperature of 101.4°F. Due to these developments, he was initially managed as a case of sepsis due to unknown etiology. Our patient received multiple transfusions of RDP, with no improvement in platelet count even after 25 units of RDP had been administered.

**Figure 1:**
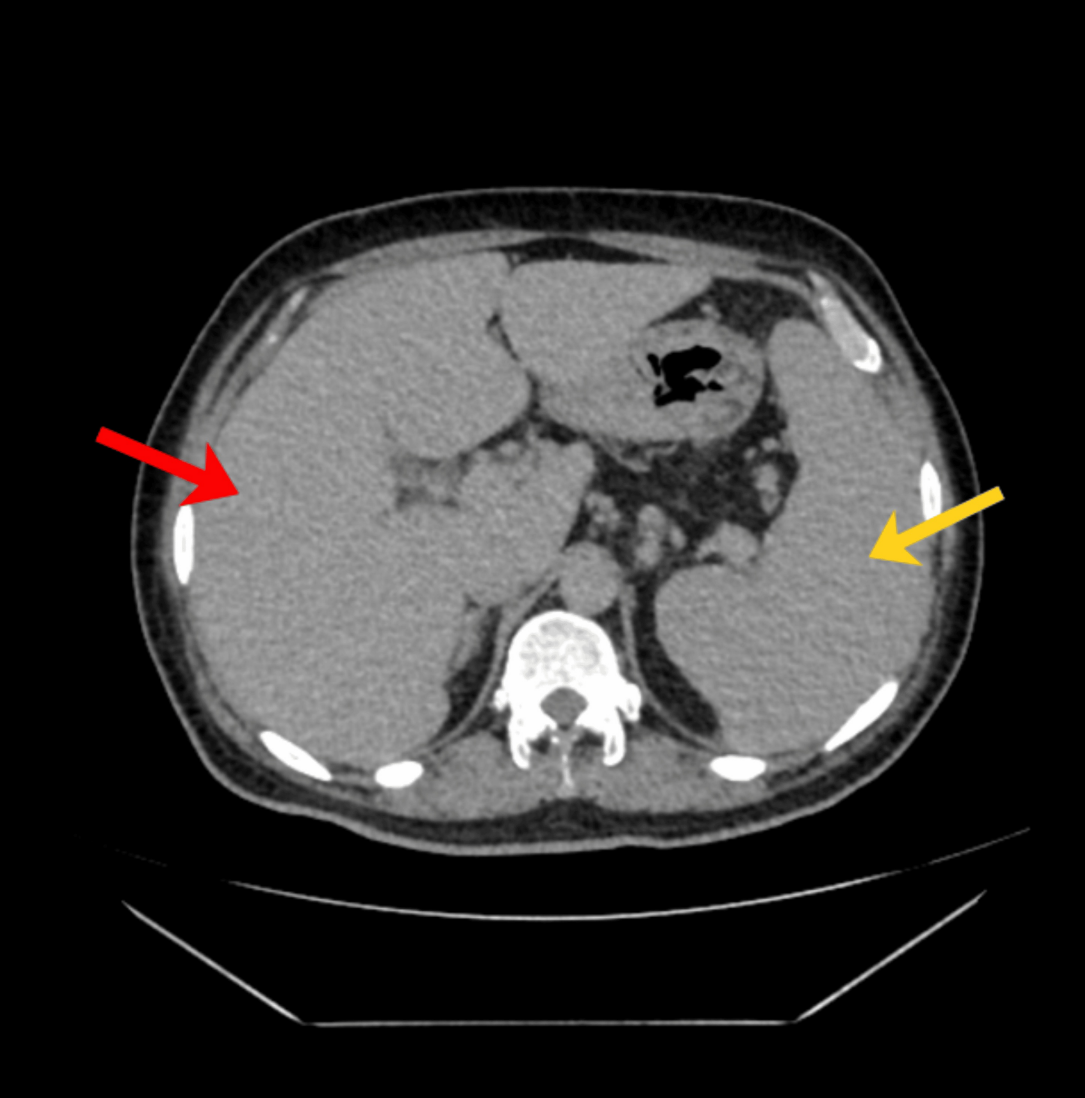
Abdomen CECT - axial view Red arrow - showing hepatomegaly (19 cm); yellow arrow - showing splenomegaly (14.7 cm).

**Figure 2:**
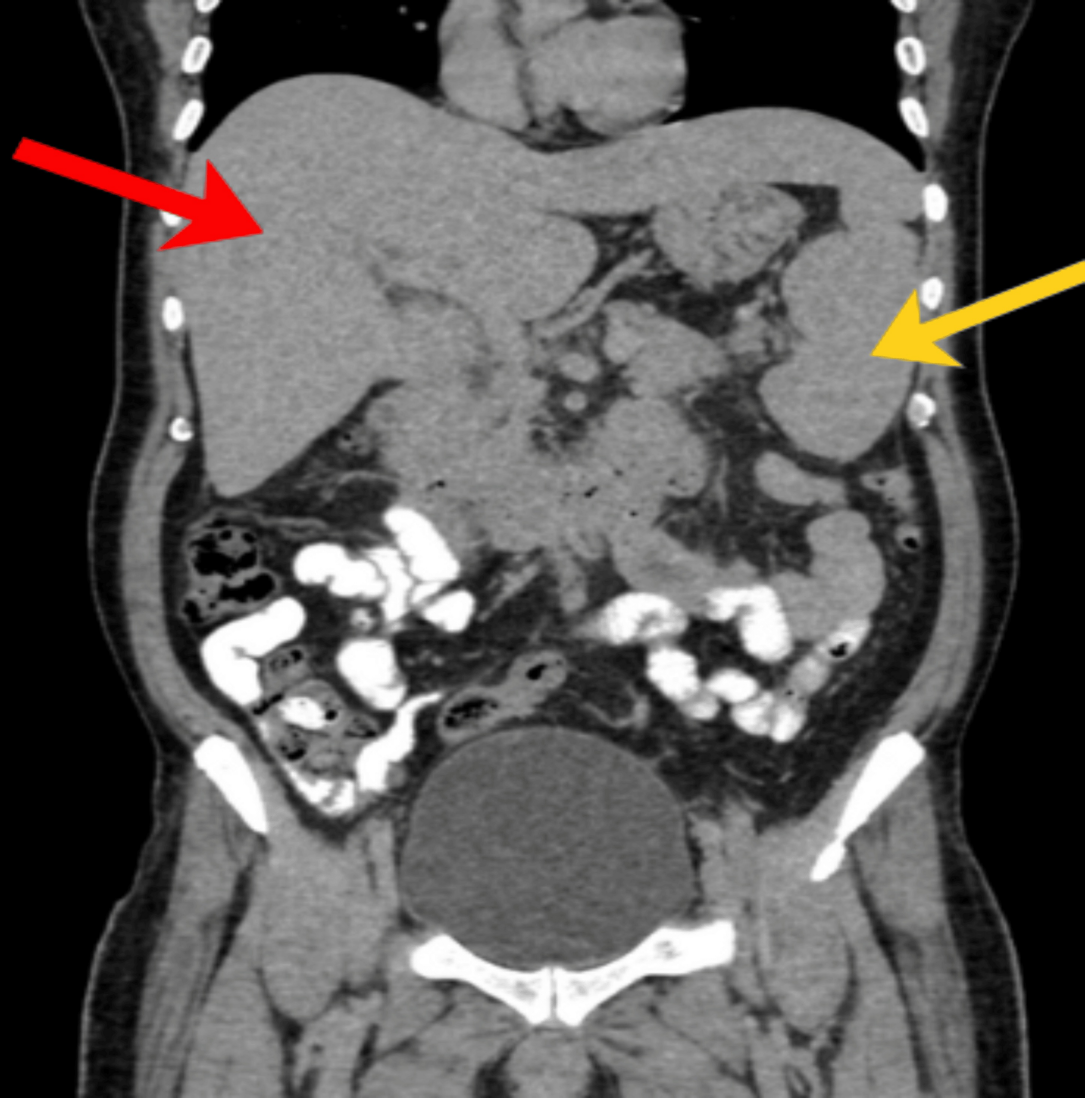
Abdomen CECT - coronal section Red arrow - showing hepatomegaly; yellow arrow - showing splenomegaly.

Serial hemograms (Table 1) revealed pancytopenia with progressive anemia (Hb declining from 14.3 to 7.6 g/dL) and severe thrombocytopenia (platelets ranging from 2,000 to 30,000/µL). Peripheral blood smear confirmed pancytopenia with increased reticulocytes. Biochemical tests (Table 2) showed elevated liver enzymes (aspartate aminotransferase (AST) up to 116 IU/L, alanine aminotransferase (ALT) 61 IU/L), high triglycerides (228 mg/dL), markedly raised serum ferritin (peak 4930 ng/mL), and mildly decreased fibrinogen (2.23 g/L). Inflammatory markers showed elevated CRP (28 mg/L). Infectious workup was negative except for positive Typhi Dot IgM, indicating acute typhoid infection. Imaging (abdomen contrast-enhanced computed tomography (CECT)) confirmed hepatosplenomegaly without ascites (Figures 1-2).

**Table 1:** Hemogram

Key hematological indices	Day 1	Day 2	Day 3	Reference range
Hemoglobin	10.1	8.3	7.6	14-18 g/dL
Platelets	10000	18000	15000	150000-400000/cu.mm
Total leukocyte count	3900	3400	4000	5000-10000/cu.mm

**Table 2:** Biochemical profile ALT - alanine aminotransferase; AST - aspartate aminotransferase; CRP - C-reactive protein.

Biochemical markers	Result	Reference range
AST	116 IU/L	0-35 IU/L
ALT	61 IU/L	4-36 IU/L
Triglycerides	228 mg/dL	40-160 mg/dL
Serum ferritin	4930 ng/mL	20-250 ng/mL
Fibrinogen	2.23 g/L	2.5-5 g/L
CRP	28 mg/L	<6 mg/L

**Figure 3:**
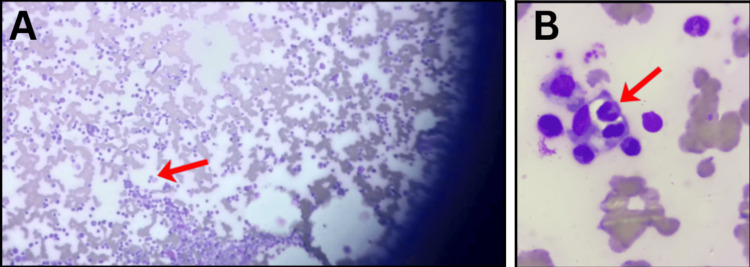
Bone marrow aspirate demonstrating hemophagocytosis (A) Macrophage with engulfed lymphocytes (red arrow) under low power magnification (100×). (B) Macrophage with engulfed lymphocytes (red arrow) under high power magnification (1000×). Reference: [3]

Bone marrow aspirate demonstrated hypercellularity with erythroid hyperplasia and evidence of hemophagocytosis, with macrophages engulfing lymphocytes (Figure 3). Coagulation studies and renal function were within normal limits. Urine culture was sterile.

**Figure 4:**
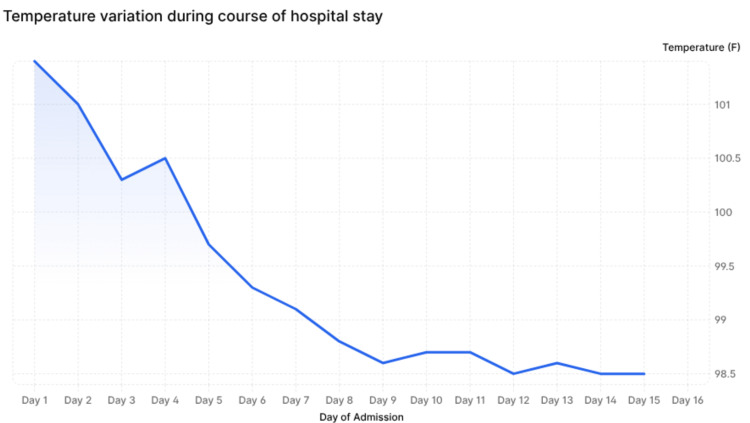
Temperature trend over the course of hospitalization

A highly elevated serum ferritin, along with elevated triglycerides, splenomegaly, normal fibrinogen, and mildly elevated C-reactive protein (CRP), skewed the diagnosis from enteric fever complicated by sepsis toward HLH secondary to typhoid infection. The pattern of temperature variation during hospital stay was as depicted in Figure 4. Several lab and clinical parameters were found to satisfy the HLH-2004 criteria (Table 3) [5] for the diagnosis of HLH, as mentioned in Table 4 (since our patient met the criteria for HLH, soluble CD25 levels and NK cell activity were not tested).

**Table 3:** HLH-2004 Diagnostic Criteria [5]

Criterion	Details
A. Molecular diagnosis	A molecular diagnosis consistent with HLH
B. Clinical/lab criteria (any five of eight required)	-
1. Fever	Temperature > 38.5°C
2. Splenomegaly	Present (length > 12 cm)
3. Cytopenia (≥2 of 3 peripheral lineages)	a. Hemoglobin < 9 g/dL
	b. Platelets < 100 × 10⁹/L
	c. Neutrophils < 1.0 × 10⁹/L
4. Hypertriglyceridemia and/or hypofibrinogenemia	a. Fasting triglycerides > 265 mg/dL
	b. Fibrinogen ≤ 1.5 g/L
5. Hemophagocytosis	In bone marrow, spleen, liver, lymph nodes, or other tissues
6. NK cell activity	Low or absent natural killer (NK) cell activity
7. Serum ferritin	≥ 500 µg/L
8. Soluble CD25 (sIL-2R)	≥ 2400 U/mL

**Table 4:** Our findings adhering to the HLH-2004 Criteria [5]

Criterion	Details	Measured values
B. Clinical/lab criteria		
1. Fever - temperature > 38.6°C	Present	38.9°C
2. Splenomegaly	Present	-
3. Cytopenia (≥ 2 of 3 peripheral lineages)	Present	-
	a. Hemoglobin < 9 g/dL	7.6 g/dL
	b. Platelets < 100 × 10⁹/L	2-30 × 10^9^/L
4. Hemophagocytosis	Present in bone marrow (Figure 3)	-
5. Serum ferritin	≥ 500 µg/L	4930 µg/L

Differential diagnosis

The presence of persistent fever, splenomegaly, cytopenias involving at least two lineages, hyperferritinemia, and bone marrow hemophagocytosis strongly supported the diagnosis of HLH per HLH-2004 criteria [5]; however, our initial differential diagnoses included sepsis-induced cytopenias, disseminated intravascular coagulation (DIC), hematological malignancies, and autoimmune cytopenias. Negative cultures and resolution of thrombocytopenia with immunosuppressants made sepsis less likely as the sole cause [6], while the absence of overt bleeding, normal fibrinogen, partial thromboplastin time, and activated partial thromboplastin time made DIC unlikely. Negative family history, absent clinical signs, and the results of the bone marrow examination ruled out other conflicting diagnoses.

Treatment

The patient received Azithromycin 1 g on day 1, followed by 500 mg/day for the next seven days [7,8]. He also received pulsed methylprednisolone therapy at 1 g/day for 5 days, followed by a tapering regimen [5]. Supportive care included close monitoring, intravenous fluids, and platelet transfusions.

Outcome and follow-up

Post-pulsed corticosteroid therapy, the patient exhibited marked clinical improvement with resolution of bleeding and stabilization of hematological parameters. At discharge, platelets had improved, and ferritin levels began to decline (1130 ng/mL after one week, 405 ng/mL after 1.5 months). He remained hemodynamically stable during follow-up visits.

## Discussion

This case demonstrates secondary HLH triggered by typhoid fever, an uncommon but recognized infectious precipitant [7,8]. The diagnosis of HLH was confirmed by fulfilling key HLH-2004 criteria: fever (initially), splenomegaly, cytopenias affecting two cell lines, elevated ferritin >500 ng/mL (measured >4900 ng/mL), hypertriglyceridemia, hypofibrinogenemia, and hemophagocytosis on bone marrow aspirate [5].

The diagnostic challenge often lies in distinguishing HLH from severe sepsis, with overlapping features such as fever, cytopenias, and organ dysfunction. However, HLH is characterized by disproportionately elevated ferritin, persistent cytopenias, and hemophagocytosis [5]. Early immunosuppressive therapy, particularly steroids, remains the cornerstone of treatment in secondary HLH, as exemplified by the patient’s rapid response. The role of etoposide and other immunomodulators is reserved for refractory or familial cases due to, in part, the adverse effect profile of such drugs [9].

Prognosis without treatment is poor, with an almost 100% mortality rate [10]. Factors, such as a hemoglobin level <9 and a platelet count <35000/µL, correlate with worse outcomes [11]. This case underlines the importance of high clinical suspicion, early biopsy, and targeted therapy. Compared to other published cases, this patient’s presentation was atypical for secondary HLH since the initial presentation was with fever and melena, and the success of corticosteroids alone highlights the variable severity and outcomes among adults.

## Conclusions

Secondary HLH, although rare, should be suspected in patients with unexplained fever, cytopenias, hepatosplenomegaly, and hyperferritinemia, especially in the setting of infections such as typhoid fever. Timely diagnosis and prompt immunosuppressive treatment can be life-saving. This case reinforces the need for clinician awareness to differentiate HLH from sepsis and other mimickers and keep it on the differential, even in a case presenting with fever and melena, which helps to initiate targeted therapy early on.
